# Reporting incidental coronary, aortic valve and cardiac calcification on non-gated thoracic computed tomography, a consensus statement from the BSCI/BSCCT and BSTI

**DOI:** 10.1259/bjr.20200894

**Published:** 2020-10-29

**Authors:** Michelle Claire Williams, Ausami Abbas, Erica Tirr, Shirjel Alam, Edward Nicol, James Shambrook, Matthias Schmitt, Gareth Morgan Hughes, James Stirrup, Ben Holloway, Deepa Gopalan, Aparna Deshpande, Jonathan Weir-McCall, Bobby Agrawal, Jonathan C L Rodrigues, Adrian J B Brady, Giles Roditi, Graham Robinson, Russell Bull

**Affiliations:** 1 University of Edinburgh/British Heart Foundation Centre for Cardiovascular Science, Edinburgh, UK; 2 Edinburgh Imaging facility QMRI, University of Edinburgh, Edinburgh, UK; 3 University Hospital Southampton, Southampton, UK; 4 Manchester University NHS Foundation Trust, Manchester, UK; 5 Departments of Cardiology and Radiology, Royal Brompton and Harefield NHS Foundation Trust, London, UK; 6 Faculty of Medicine, National Heart and Lung Institute, Imperial College, London, UK; 7 Plymouth Hospitals NHS Trust, Derriford Road, Plymouth, UK; 8 Royal Berkshire Hospital NHS Foundation Trust, Craven Road, Reading, UK; 9 Queen Elizabeth Hospital, Birmingham, UK; 10 Imperial College London & Cambridge University Hospital, Cambridge, UK; 11 Glenfield Hospital, University Hospitals of Leicester, Leicester, UK; 12 University of Cambridge School of Clinical Medicine, Cambridge, UK; 13 Royal Papworth Hospital NHS Foundation Trust, Cambridge, UK; 14 Royal United Hospitals Bath NHS Foundation Trust, Bath, UK; 15 Glasgow Royal Infirmary, 16 Alexandra Parade, Glasgow, UK; 16 University of Glasgow, University Avenue, Glasgow, UK; 17 Royal Bournemouth Hospital, Castle Lane East, Bournemouth, UK

## Abstract

Incidental coronary and cardiac calcification are frequent findings on non-gated thoracic CT. We recommend that the heart is reviewed on all CT scans where it is visualised. Coronary artery calcification is a marker of coronary artery disease and it is associated with an adverse prognosis on dedicated cardiac imaging and on non-gated thoracic CT performed for non-cardiac indications, both with and without contrast. We recommend that coronary artery calcification is reported on all non-gated thoracic CT using a simple patient-based score (none, mild, moderate, severe). Furthermore, we recommend that reports include recommendations for subsequent management, namely the assessment of modifiable cardiovascular risk factors and, if the patient has chest pain, assessment as per standard guidelines. In most cases, this will not necessitate additional investigations. Incidental aortic valve calcification may also be identified on non-gated thoracic CT and should be reported, along with ancillary findings such as aortic root dilation. Calcification may occur in other parts of the heart including mitral valve/annulus, pericardium and myocardium, but in many cases these are an incidental finding without clinical significance.

## Introduction

Cardiovascular disease is one of the leading causes of death in the western world, with coronary artery disease accounting for >10% of all deaths in the UK in 2017.^1^ The burden of cardiovascular disease in patients investigated for non-cardiovascular disease is high, with almost the same number of deaths attributable to cardiovascular disease as lung cancer in the National Lung Screening Trial.^2^ Identification of coronary artery disease enables early intervention targeted against modifiable risk factors, which can significantly reduce future coronary events.^3^ Diagnostic tools that provide further opportunities to detect coronary artery disease, therefore, have the potential to reduce the burden of associated morbidity and mortality.

Given the >5.5 million CT scans were performed in the UK in 2018–19, we estimate that ~950 000 thoracic CT scans are performed annually in the UK.^4^ A significant increase in the number of low-dose chest CT scans in the UK is expected with the expansion of the Targeted Lung Health Checks Program from 2020.^5^ In patients undergoing lung cancer screening, one-third of patients are at a high cardiovascular risk but are not taking statin therapy.^6^ The heart is an important component of all imaging involving the chest. Cardiac pathology may be asymptomatic or provide an explanation for the patient’s presentation. Cardiac and respiratory diseases share risk factors, such as smoking, and pathogenic mechanisms, such as inflammation. Furthermore, review of the heart may identify cardiac sequelae of lung disease.

**Figure 1.**
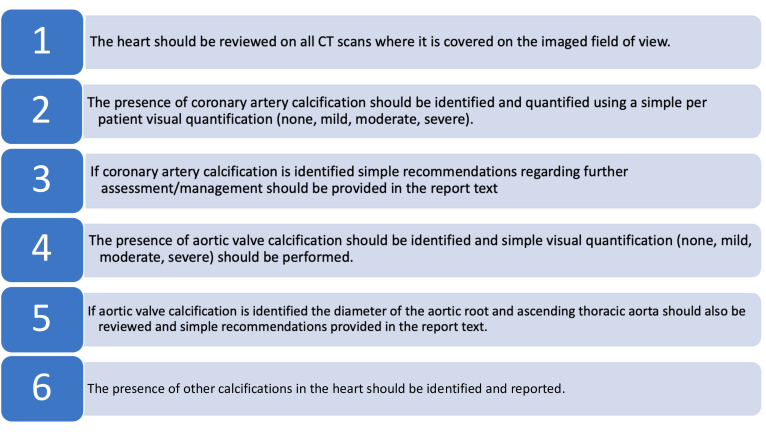
Recommendations for the assessment of cardiac calcification on routine thoracic CT

**Figure 2.**
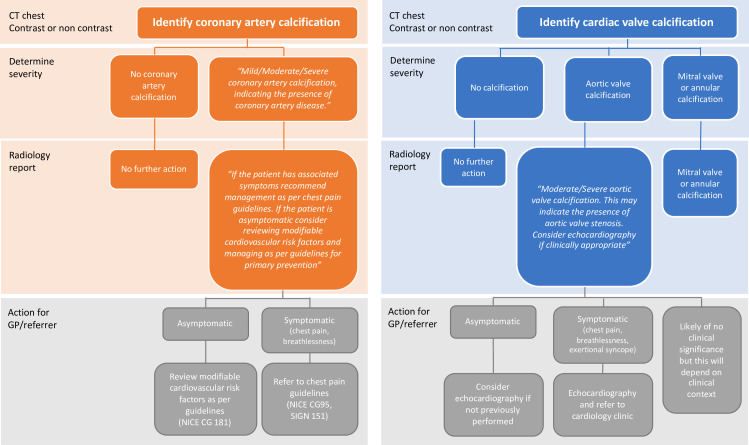
Flow diagram for the assessment of coronary artery calcification and cardiac valve calcification on routine thoracic CT.

The aim of this joint guideline from the British Society of Cardiovascular Imaging/British Society of Cardiac Computed Tomography (BSCI/BSCCT) and British Society of Thoracic Imaging (BSTI) is to provide guidance for radiologists regarding the reporting of incidental coronary and cardiac calcification on routine thoracic CT performed for non-cardiac indications without electrocardiogram (ECG) gating (Figures 1 and 2). In particular, we recommend the reporting of coronary artery calcification (CAC) when visualised on all CT scans. We also provide guidance on how to classify the severity of CAC on a per patient basis and aim to increase the awareness of the prognostic implications of CAC.

Recommendation 1: the heart should be reviewed on all CT scans where it is covered on the imaged field of view.

## Coronary artery calcification

CAC can be identified as high attenuation material in the path of a coronary artery. CAC is an established biomarker for the burden of atherosclerosis^7,8^ with an increase in CAC associated with increased risk of cardiovascular events in symptomatic and asymptomatic patients.^9–16^ It is not a method to identify the severity of coronary artery stenoses, and significant CAC may be present in the absence of flow-limiting coronary artery stenoses. Furthermore, coronary artery disease may be present in the absence of CAC. A high burden of CAC may be present in patients classified as “low risk” by traditional risk factor scoring systems and conversely absent in patients classified at “high risk”. Combining CAC with traditional risk factors can therefore improve coronary artery disease risk stratification^10,17^ and appropriately target statin therapy.^18^ In addition to the early detection of coronary artery disease, the visualisation of CT coronary artery plaque by patients has been shown to improve adherence with lifestyle modifications and medications.^19–21^

CAC is formally evaluated using dedicated non-contrast ECG-gated cardiac CT, performed with 3 mm contiguous slices and a tube voltage of 120 kVp. The Agatston scoring system is the most widely used method to assess CAC, although alternatives such as the mass and volume scores are available.^22^ Agatston scoring is performed using semi-automated software to identify areas of calcification (above 130 Hounsfield units), which are then weighted based on the maximum attenuation density and summed.^23^ Patients may then be classified into risk groups, with CAC score 0 Agatston units (AU) (very low risk), 1–99 AU (low risk), 100–299 AU (moderate risk), and ≥300 AU (high risk).^24^ Asymptomatic patients with an Agatston score >300 AU have a sevenfold increase in the risk of myocardial infarction or coronary heart disease death compared to patients with no CAC.^10^

### Coronary artery calcification on non-gated thoracic CT

CAC can be identified on non-gated thoracic CT with an excellent diagnostic accuracy compared to gated CT.^25,26^ However, CAC is frequently not reported on non-gated thoracic CT,^27–30^ and a recent survey demonstrated only 17% of non-cardiothoracic radiologists in Canada were aware of the correlation between CAC scores on gated and non-gated thoracic CT.^31^ The incidence of CAC on non-gated thoracic CT performed for non-cardiac indications varies from 26 to 93% depending on the population assessed.^28–30,32–34^ It is associated with poorer prognosis in a variety of patient cohorts including patients with chronic obstructive pulmonary disease, pulmonary embolism, cancer and in unselected patients undergoing thoracic CT.^32,35–39^ In the National Lung Screening Trial, CAC was associated with an increased risk of coronary artery disease-related death, with a CAC score of 100–1000 AU associated with a fourfold increase, and CAC score of >1000 AU associated with a sevenfold increase compared to patients without CAC.^35^

### Reporting coronary calcification

While conventional Agatston scoring remains the gold-standard assessment, it requires dedicated software and training. A number of semi-quantitative ordinal scoring systems have been developed on a per segment, per vessel or per patient basis.^35,40,41^ These correlate well with Agatston scoring and their prognostic utility have been established.^35,41,42^ However, these semi-quantitative ordinal scoring systems also take time and training to perform.

**Figure 3.**
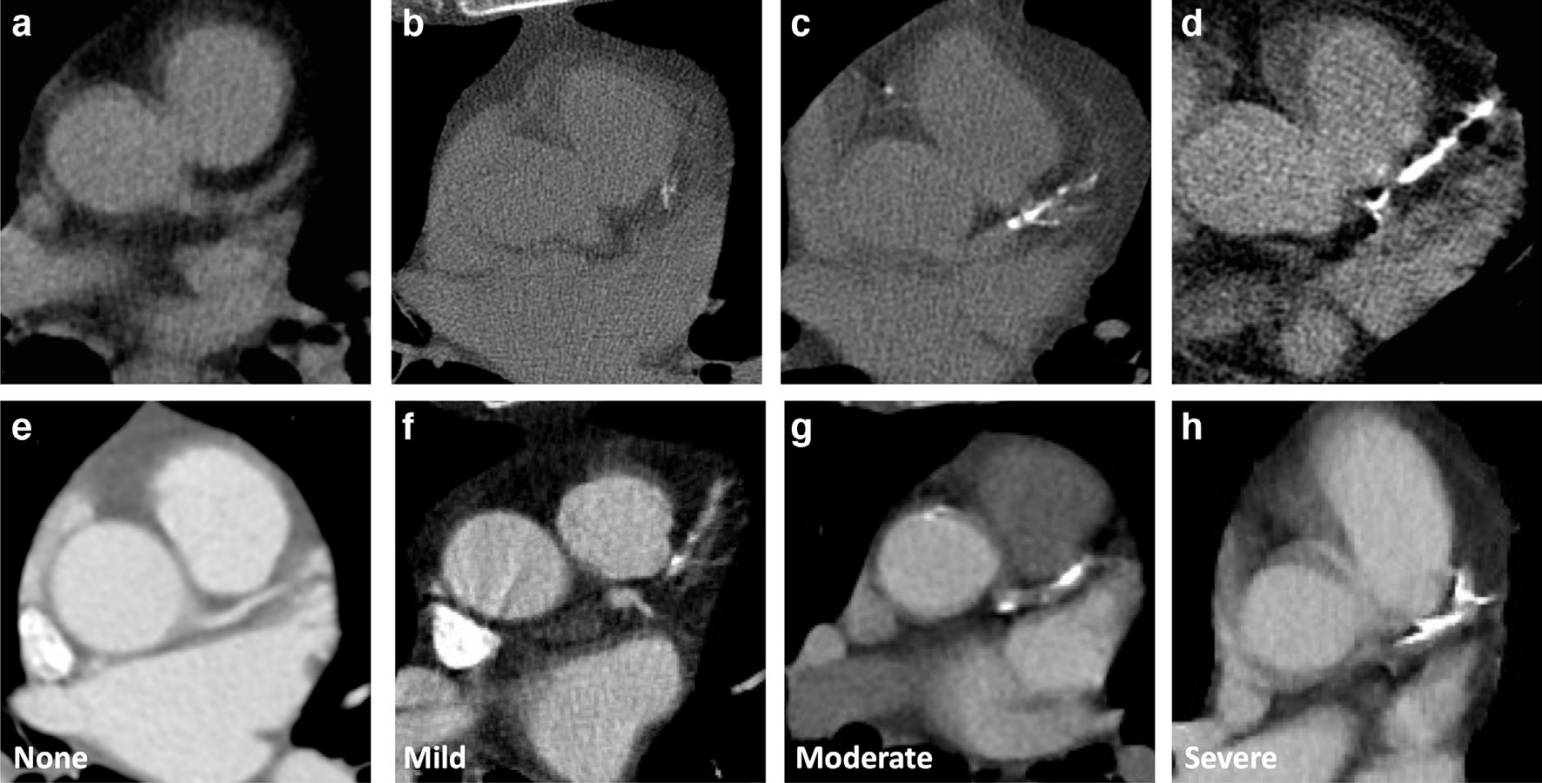
Coronary artery calcification severity. Images show different severity of coronary calcification from different patients on non-contrast (A–D) and contrast enhanced CT (E–H). Images show no coronary artery calcification (A, E) and mild (B, F), moderate (C, G) and severe (D, H) coronary artery calcification.

For non-gated thoracic CT in routine clinical practice, we therefore recommend a simple visual ordinal score performed on a whole patient basis. CAC is scored as None, Mild, Moderate or Severe on a whole patient basis, aiming to summarise the cumulative findings in all the coronary arteries (Figure 3, Supplementary Material 1). This can be applied to both non-contrast and contrast-enhanced images. It provides some stratification of patients into risk groups, while being rapid and easy to perform in clinical practice. In the National Lung Screening Trial the application of a per patient visual assessment identified patients at increased risk of subsequent coronary heart disease-related death, with good correlation with Agatston scoring, excellent interobserver agreement and acceptability to non-cardiac radiologists.^35^

Supplementary Material 1.

Recommendation 2: the presence of coronary artery calcification should be identified and quantified using a simple per patient visual quantification (none, mild, moderate, severe).

Recommendation 3: if coronary artery calcification is identified simple recommendations regarding further assessment/management should be provided in the report text.

CAC is a frequent finding on thoracic CT and is not necessarily an indication for further imaging or referral to a cardiologist. Instead, review of the clinical features and cardiovascular risk factors is recommended, usually by the general practitioner or referring physician. If patients are symptomatic with suspected coronary artery disease. they should be managed as per standard guidelines (*e.g.* NICE CG95 2016 revision or SIGN 151). If they are asymptomatic, we recommend the referrer or general practitioner review and consider modifiable cardiovascular risk factors and manage these as per standard guidelines (*e.g.* NICE CG 181). For asymptomatic patients, there is no current evidence to support further imaging (ischaemia testing, CT coronary angiography or invasive coronary angiography).

It is important to remember that the request for imaging will often contain limited ancillary information regarding other pathologies or investigations. We, therefore, recommend that information regarding CAC is provided to the referring clinician so that they can take this into consideration with the overall management of the patient.

We do not recommend an upper age limit for the reporting of CAC, nor changing how CAC is reported based on age. The term “normal for age” should be avoided, as the risk of cardiovascular events increases in proportion to the amount of CAC in all age groups. In young patients (<40 years old), the presence of severe CAC is unusual, and the assessment of cardiovascular risk factors or symptoms is particularly important. Similarly, the presence of malignancy is not a reason to ignore CAC, as patients with malignancy are at a similar or increased risk of cardiac events secondary to the disease process and cardiotoxic therapies. If previous coronary intervention is apparent on imaging, such as the presence of coronary artery stents or coronary artery bypass grafts, then assessment of incidental CAC is not required as the presence of coronary artery disease has already been established.

Suggested text that may be included in the summary of the report is as follows (Automatic insertion of dictation codes can be used to speed this process, Table 1, Supplementary Material 1.

“Mild/Moderate/Severe coronary artery calcification, indicating the presence of coronary artery disease. If the patient has associated symptoms recommend management as per chest pain guidelines (*e.g.* NICE CG95, SIGN 151). If the patient is asymptomatic consider reviewing modifiable cardiovascular risk factors and managing as per guidelines for primary prevention (*e.g.* NICE CG 181).”

**Table 1.** Reporting recommendations for coronary artery and aortic valve calcification

	Suggested report text
Coronary artery calcification	Mild/Moderate/Severe coronary artery calcification, indicating the presence of coronary artery disease. If the patient has associated symptoms recommend management as per chest pain guidelines (*e.g.* NICE CG95, SIGN 151). If the patient is asymptomatic consider reviewing modifiable cardiovascular risk factors and managing as per guidelines for primary prevention (*e.g.* NICE CG 181).
Aortic valve calcification	Moderate/Severe aortic valve calcification. This may indicate the presence of aortic valve stenosis. Consider echocardiography if clinically appropriate.

## Aortic valve calcification

Aortic valve disease is the most common cardiac valve disease in the developed world, with moderate or severe aortic stenosis reported in ~5% of patients aged >75 years.^43,44^ The leading cause of aortic stenosis is calcific valvular degeneration, although underlying bicuspid aortic valve (BAV) disease is an important consideration in younger patients.^43,44^ Aortic valve calcification on CT is associated with the severity of aortic stenosis assessed using echocardiography^45–47^ and cut-off values for the aortic valve Agatston calcium score have been established to identify patients with severe stenosis (≥2065 AU for males and ≥1274 AU for females).^48,49^ Aortic valve calcification is also associated with the speed of disease progression and an increased risk of adverse events, including valve replacement and mortality.^45,50,51^ Aortic valve calcification is of particular utility in cases of low-flow low-gradient aortic stenosis where echocardiographic assessment can be challenging.^52^ Consequently, the assessment of aortic valve calcification on ECG-gated cardiac CT is now part of the 2017 ESC/EACTS valvular heart disease guidelines.^53^ The clinical importance of mild aortic valve calcification is currently uncertain, and further evidence is required in this area. An exception to this however is patients with bicuspid aortic valves, where significant aortic stenosis can occur in the presence of mild aortic valve calcification.

### Aortic valve calcification on non-gated thoracic CT

**Figure 4.**
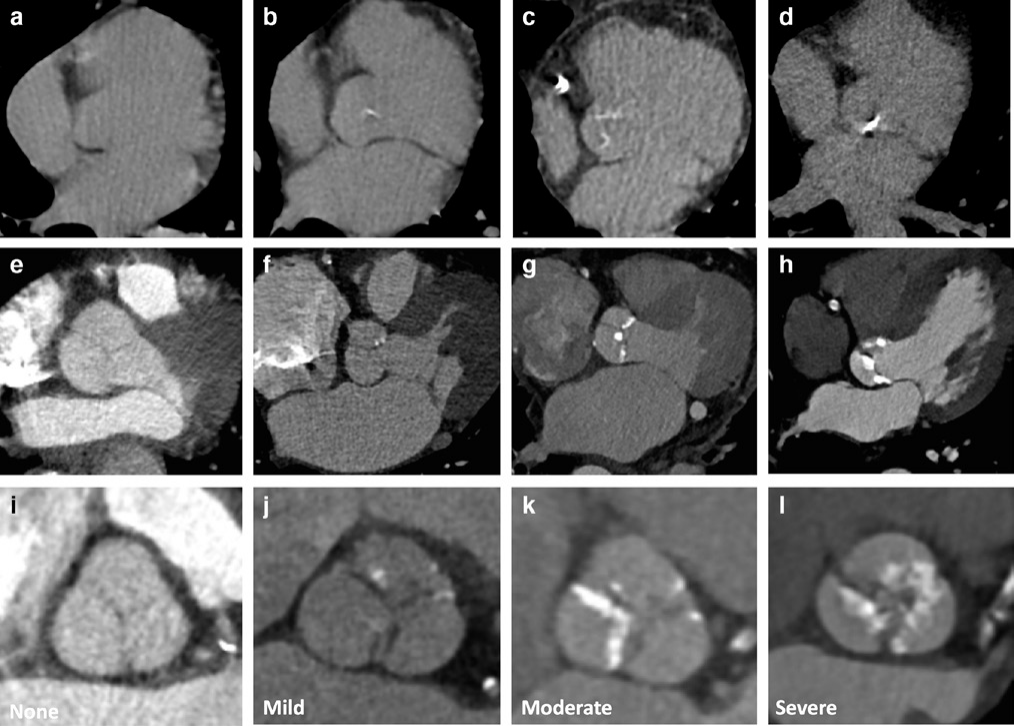
Aortic valve calcification severity. Images show different severities of aortic valve calcification from different patients on non-contrast (A–D) and contrast enhanced CT (E–L). Multiplanar reformats can help to differentiate aortic valve calcification from annular or aortic calcification (I–L). Images show no calcification (A, E, I) and mild (B, F, J), moderate (C, G, K) and severe (D, H, L) aortic valve calcification.

Calcification of the aortic valve can be identified as high attenuation material in the region of the aortic valve (Figure 4, Supplementary Material 1). Care should be taken to differentiate aortic valve calcification from calcification in the aortic root, mitral annulus or coronary arteries. The severity of aortic valve calcification on non-gated thoracic CT correlates with echocardiographic assessment of aortic stenosis.^54–56^ It is a common incidental finding, with its frequency depending on the age of the population and indication for imaging.^55,57,58^ However, the presence of aortic valve calcification is frequently not reported.^57^

### Reporting aortic valve calcification

Agatston scoring of aortic valve calcification can be performed on non-gated CT, with good correlation with echocardiographic parameters.^46,59^ However, this is time consuming, and requires dedicated software and training. We therefore recommend a simple visual ordinal assessment, where aortic valve calcification is described as None, Mild, Moderate or Severe (Figure 4). This is quick to perform and can be performed on contrast and non-contrast images. It also correlates well with formal Agatston calcium scoring and with echocardiographic findings.^54,55^

The presence of moderate or severe aortic valve calcification may identify patients who require further assessment such as echocardiography. It may also be the cause of presenting symptoms such as dyspnoea. However, in many cases the presence of aortic valve disease will already be known, and echocardiography may have already been performed. For patients with aortic valve calcification, the diameter of the aortic root and ascending aorta should also be reviewed, on multiplanar reformats where possible, as post-stenotic dilation may be apparent even on non-contrast imaging. This is not an immediate indication for recall for contrast enhanced imaging, but should prompt further assessment of the severity of the aortic stenosis and further assessment of aortic anatomy may be required.

Suggested text that can be included in the summary of the report is as follows (Automatic insertion of dictation codes can be used to speed this process, Table 1):

“Moderate/Severe aortic valve calcification. This may indicate the presence of aortic valve stenosis. Consider echocardiography if clinically appropriate.”

Recommendation 4: the presence of aortic valve calcification should be identified and visual quantification (none, mild, moderate, severe) should be performed.

Recommendation 5: if aortic valve calcification is identified the diameter of the aortic root and ascending thoracic aorta should also be reviewed and simple recommendations provided in the report text.

## Mitral, myocardial and pericardial calcification

**Figure 5.**
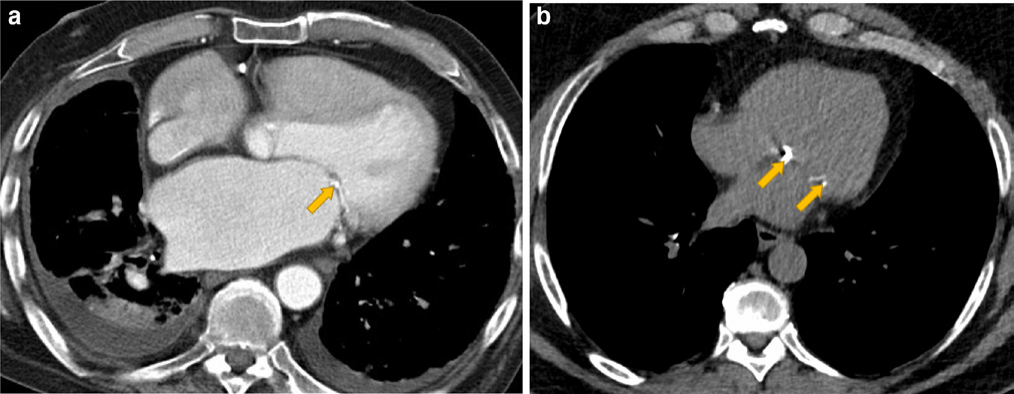
Mitral leaflet and mitral annular calcification. Contrast enhanced CT (A) shows mitral leaflet calcification (A, yellow arrow) in a patient with mitral valve dysfunction and associated enlarged left atrium and pleural effusions. In comparison the non-contrast CT (B) shows mitral annular calcification (yellow arrows) without clinical sequelae.

**Figure 6.**
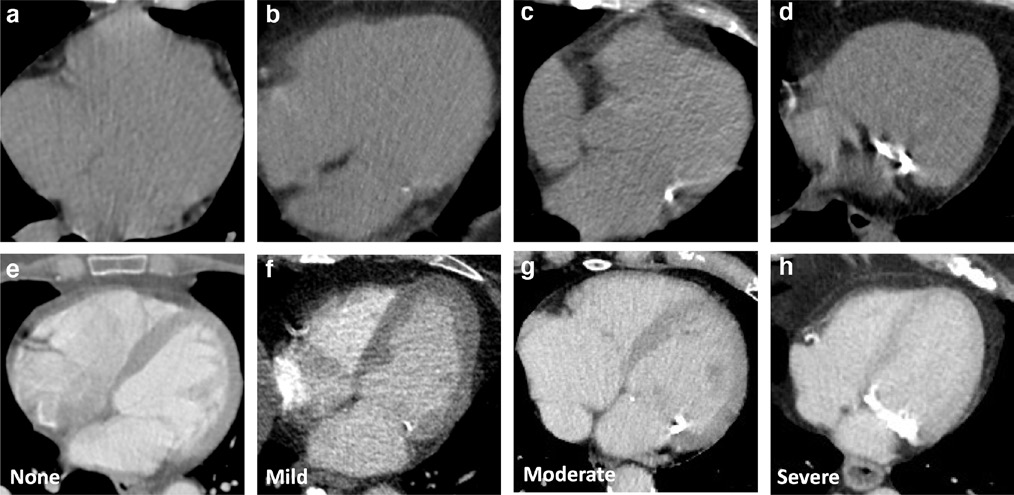
Mitral calcification severity. Images show different severity of mitral calcification from different patients on non-contrast (A–D) and contrast CT (E–H). Images show no calcification (A, E) and mild (B, F), moderate (C, G) and severe (D, H) calcification.

Calcification of the mitral leaflets or mitral annulus is a common incidental finding, occurring on approximately 8% of routine thoracic CT.^60^ Mitral leaflet calcification is uncommon and may be associated with rheumatic heart disease or advanced renal impairment. It is usually subtle and limited to the leaflet tips, whereas mitral annular calcification can be extensive, usually demonstrating a curvilinear morphology in the posterior and outer ring of the valve (Figure 5). Patients with mitral leaflet calcification may require further assessment of mitral valve function whereas mitral annular calcification is rarely a therapeutic target. It is also important not to mistake mitral annular calcification for calcification in the left circumflex coronary artery. Mitral calcification can be graded on non-contrast and contrast-enhanced CT using a simple visual grading of None, Mild, Moderate or Severe (Figure 6).

**Figure 7.**
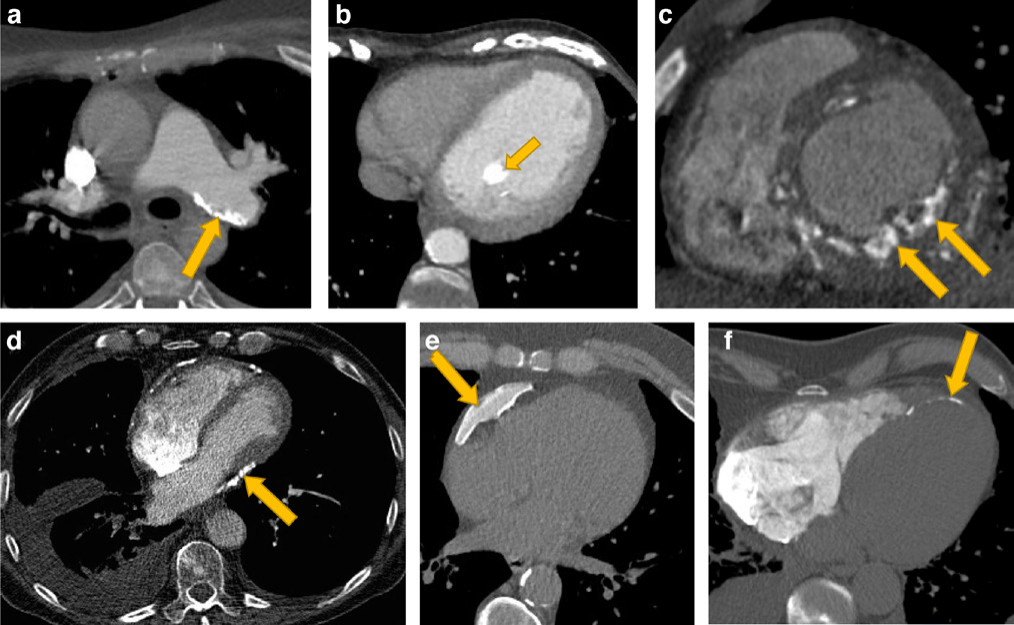
Examples of cardiac calcification in other sites including (A) pulmonary arteries, (B) papillary muscle, (C, F) myocardium and (D, E) pericardium. Image (C) shows myocardial calcification in renal failure and (F) shows myocardial calcification in an infarct. (E) shows an example of benign pericardial calcification, whereas (D) shows pericardial calcification associated with constrictive pericarditis with associated atrial enlargement, tubular ventricular morphology, pleural effusion and a distended inferior vena cava.

Myocardial calcification (Figure 7) usually arises secondary to myocardial infarction, although other less common aetiologies include trauma, inflammation, neoplastic infiltration, hypertrophic cardiomyopathy or infection. Dystrophic myocardial calcification associated with infarction has a thin curvilinear appearance, with associated myocardial thinning or fatty infiltration. Calcification may also occur in the papillary muscles (Figure 7). Patients with chronic rheumatic mitral stenosis may also demonstrate left atrial wall calcification and patients with prior pulmonary vein ablation can develop calcification at the ablation sites. Furthermore, unusual calcification patterns may occur in patients with adult congenital heart disease, and a clinical history will be of required to interpret this. The significance of myocardial calcification will depend on the clinical context and indication for imaging.

Pericardial calcification (Figure 7) usually deposits at sites of previous pericardial inflammation or fibrosis. Conditions associated with pericardial calcification include infection (especially viral and tuberculosis), cardiac surgery, trauma, radiotherapy, rheumatic heart disease, collagen vascular disease, uraemic pericarditis and haemopericardium. Calcified pleural plaques on the mediastinal reflection should not be confused for pericardial calcification. The significance of pericardial calcification will depend on the clinical context. If ancillary features of constrictive pericarditis are identified (Figure 7), then this may warrant further investigation.

Recommendation 6: the presence of other calcifications in the heart should be identified and reported.

## Conclusion

The identification of calcification in the coronary arteries can provide information on the presence of previously unknown coronary artery disease and trigger an assessment of cardiovascular risk factors or associated symptoms such as chest pain. The identification of aortic valve calcification may identify patients with previously unknown aortic valve disease. Calcification in other parts of the heart including the mitral valve, myocardium and pericardium may also be identified on thoracic CT. These are often asymptomatic and the significance will depend on the clinical context.

These guidelines provide recommendations for the reporting of coronary, aortic valve and other cardiac calcification identified on non-gated thoracic CT performed for non-cardiac indications. The guidelines are simple and designed for routine clinical use for all radiologists reporting thoracic CT. The use of simple visual ordinal scoring systems for coronary and aortic valve calcification are designed to minimise the time required to implement these guidelines and provide a standardised reporting framework that is easily understood by reporters and referrers.
