# Supplementary material for: Reporting incidental coronary, aortic valve and cardiac calcification on non-gated thoracic computed tomography, a consensus statement from the BSCI/BSCCT and BSTI
Source: Br J Radiol. 2020 Oct 29;94(1117):20200894. doi: 10.1259/bjr.20200894 (PMC7774698; doi:10.1259/bjr.20200894)
Supplement: Supplementary Material 1. [file bjr.20200894.suppl-01.docx]

**Reporting incidental coronary, aortic valve and cardiac calcification on non-gated thoracic computed tomography, a consensus statement from the BSCI/BSCCT and BSTI**

**Supplementary information**

*Supplementary Figure 1*

Axial CT images from a 43-year old male with metastatic bronchogenic carcinoma showing a soft tissue mass (thin arrow) and mediastinal lymphadenopathy (arrowhead). There is incidental mild coronary artery calcification in the left anterior descending coronary artery (thick arrow). The patient was a heavy smoker with a 20 pack year history.

*
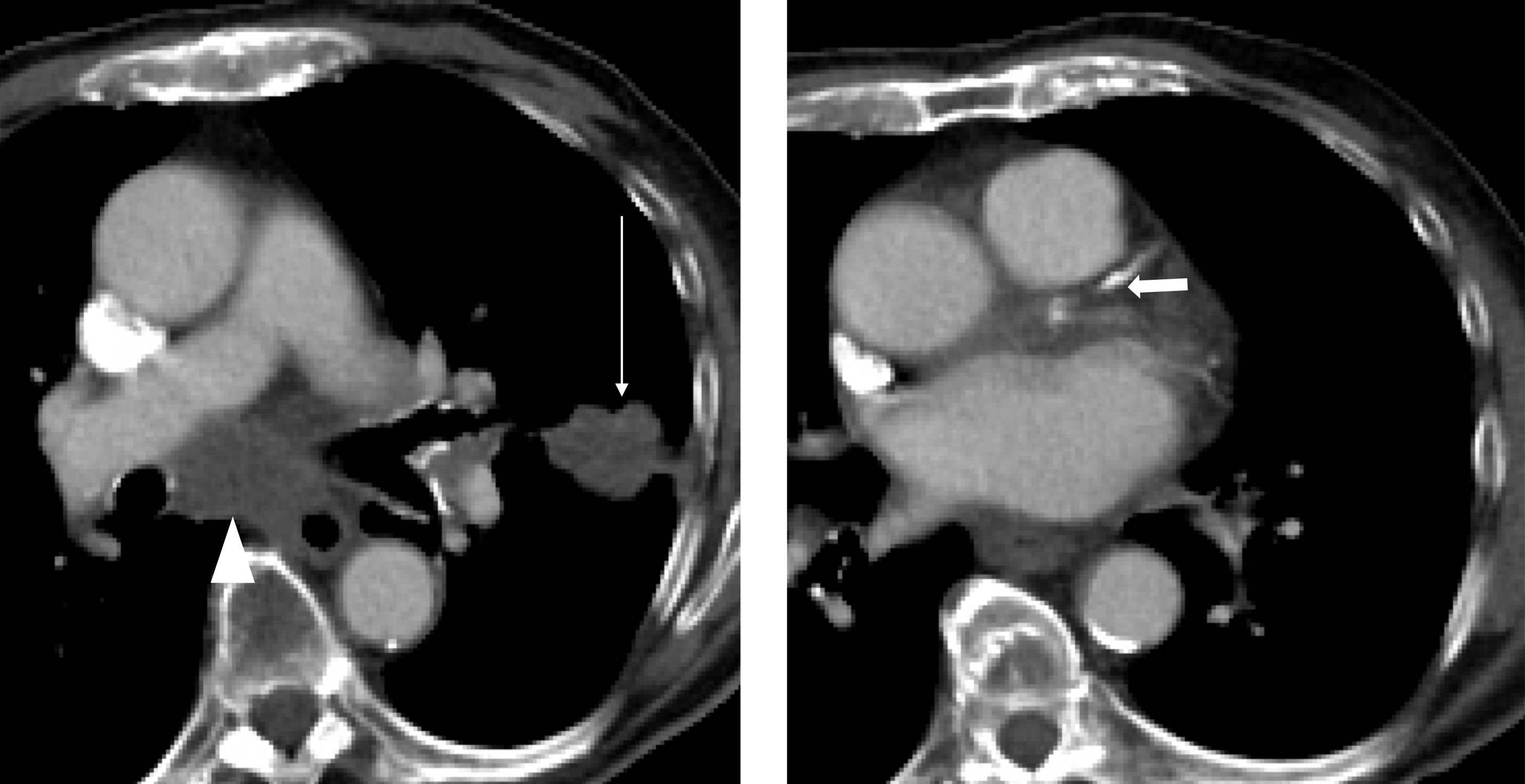
*

*Supplementary Figure 2*

Axial CT image from a 55-year old male with known rheumatoid arthritis being investigated for dyspnea. There is subtle subpleural reticulation at the lung bases (block arrows) and incidental mild LAD coronary calcification (arrowhead).


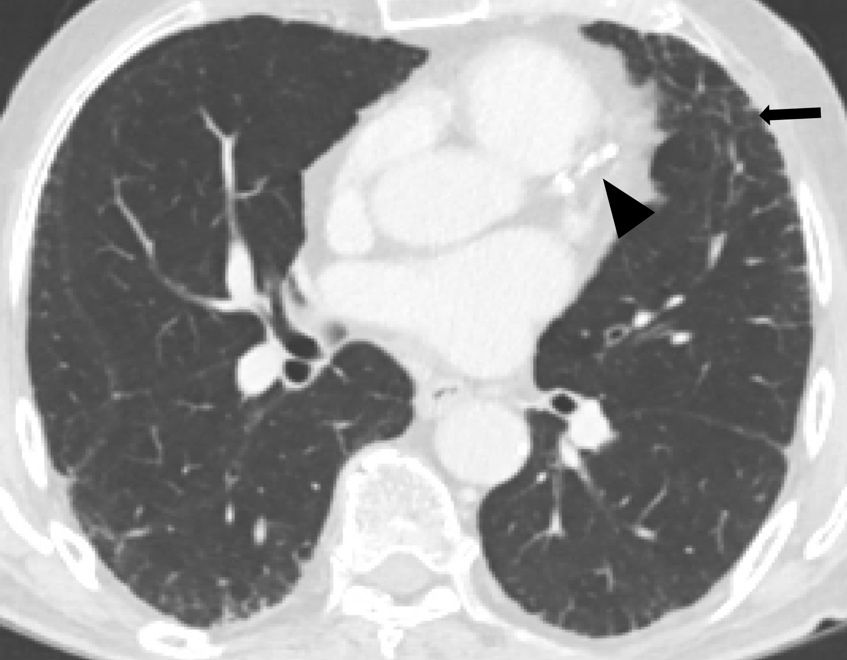


*Supplementary Figure 3*

Axial CT images from a 67-year old male with known asbestos related pleuro-parenchymal (not shown) & pericardial disease (thin arrows). There is moderate LAD calcification (thick arrow) that shows interval progression on these three serial annual surveillance scans performed for follow-up of asbestosis.


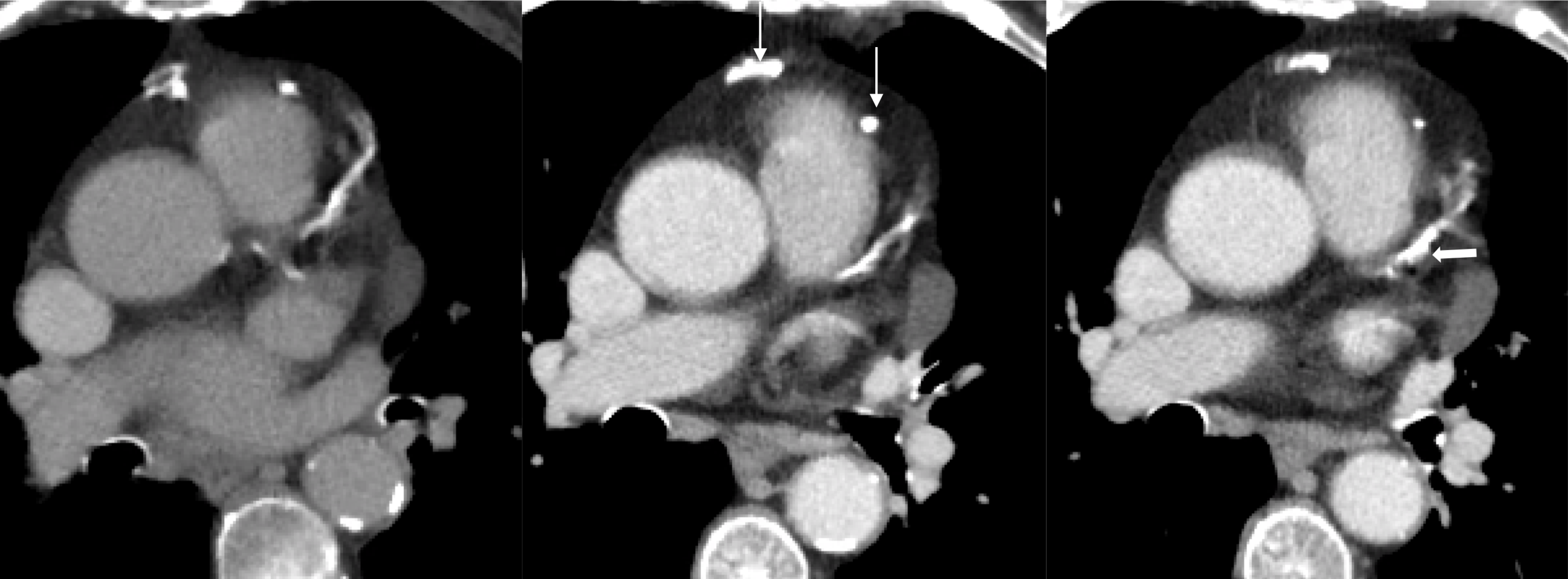


*Supplementary Figure 4*

Axial CT image from a 60-year old male smoker who had a non-ECG gated CT thorax for investigation of dyspnea. There was no discernible abnormality in the lungs or pleura but incidental calcification was noted in the RCA (thick arrow) as well as calcification in the aortic valve (thin arrow) and mitral valve (arrowhead).


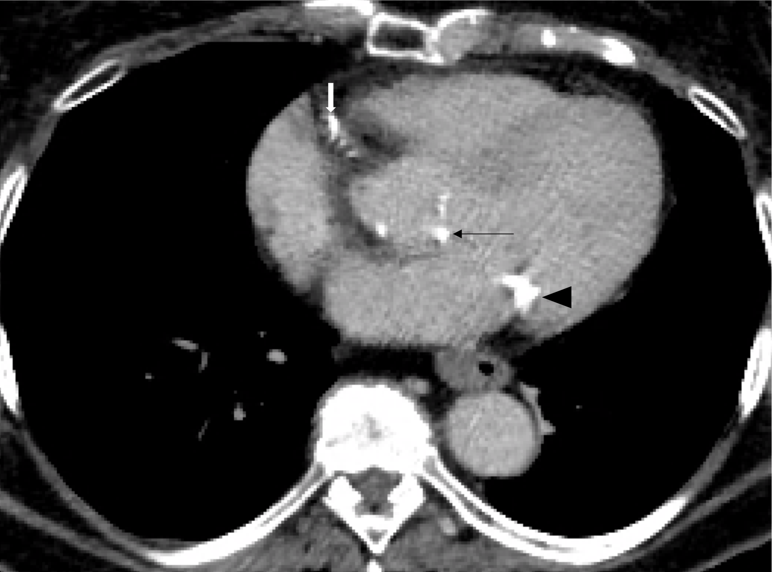


*Supplementary Figure 5*

Non-contrast axial thoracic CT images from a 57-year old male with left lower lobe consolidation and severe coronary calcification. Severe diffuse coronary artery calcification is seen in the left circumflex (thin arrow), left anterior descending coronary artery (arrowhead) and right coronary artery (thick arrow)


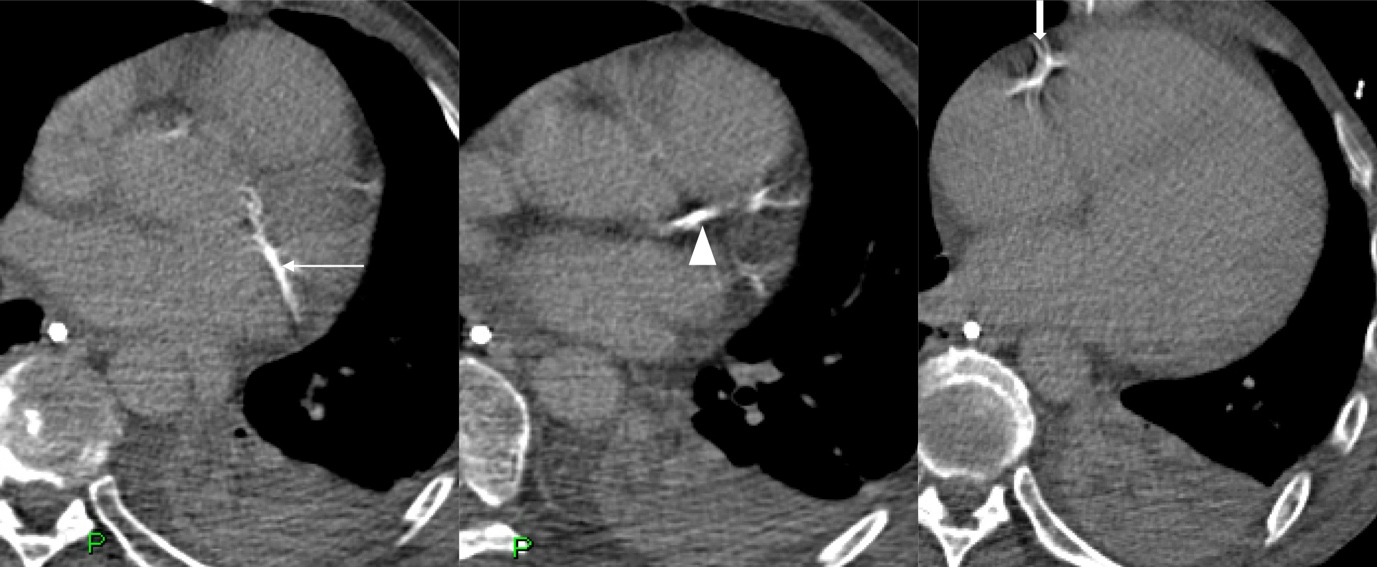


*Supplementary Figure 6*

Axial CT images from 52-year old female on dialysis secondary to renal failure. Two thoracic CT scans (one non-contrast [A, B, C] and the other with contrast [D]) taken a month apart. These show diffuse severe three vessel coronary artery calcification, with calcification in the right coronary artery (A and B), left circumflex (C) and left mainstem and left anterior descending coronary artery (D).


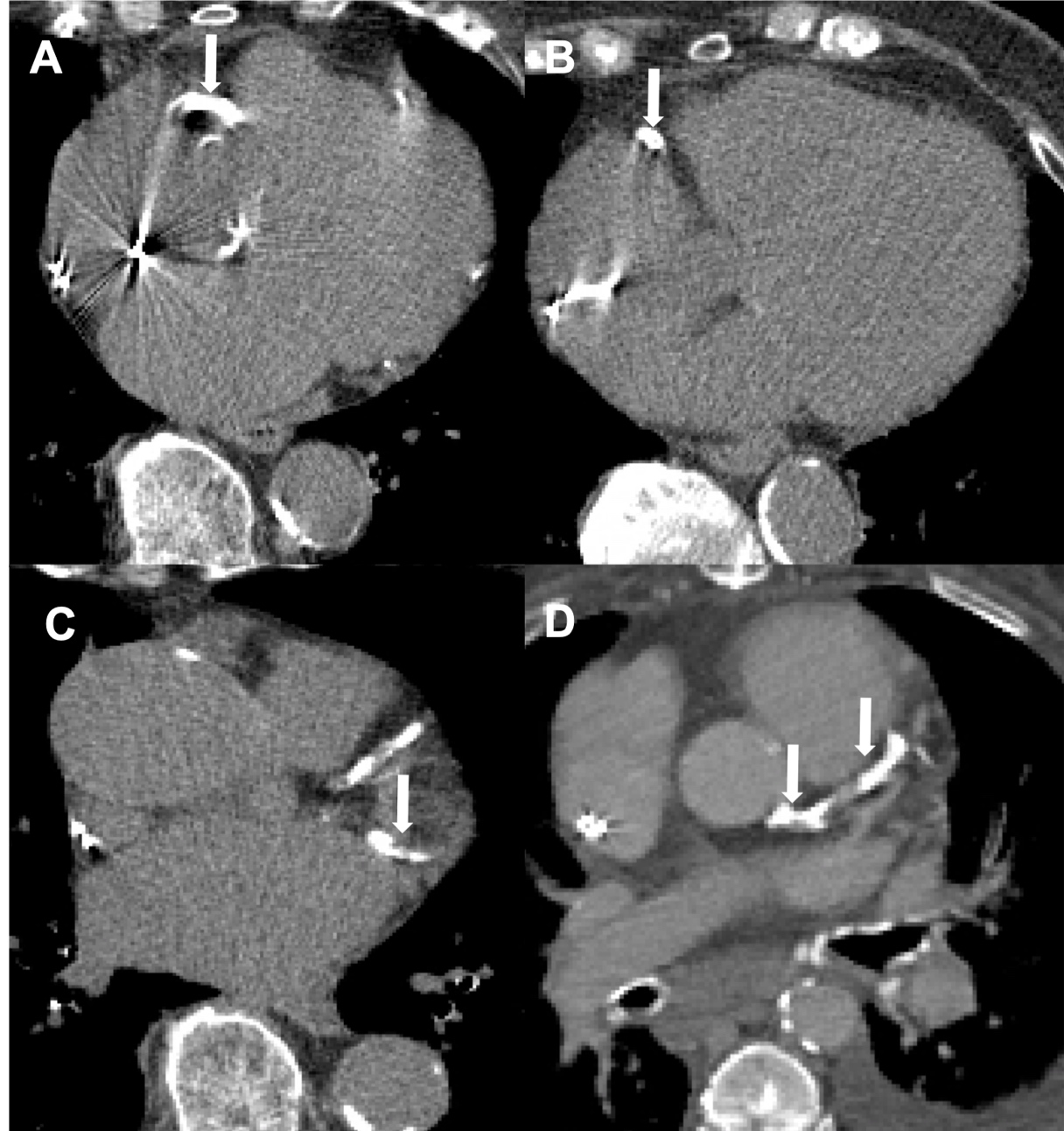


*Supplementary Table 1*

Exemplar radiology report for a non-contrast computed tomography (CT) chest performed for query bronchiectasis, which is normal apart from the presence of moderate coronary artery calcification.

| *Non contrast CT chest.*  *No previous images for comparison.*  *Normal lung parenchyma. Normal pleural and pericardial spaces. No enlarged mediastinal or axillary lymph nodes. Moderate coronary artery calcification. The partially imaged upper abdominal viscera are unremarkable. No significant bony abnormalities.*  *Conclusion*  *Normal lung parenchyma.*  *Moderate coronary artery calcification, indicating the presence of coronary artery disease.*  *(If the patient has associated symptoms recommend management as per chest pain guidelines (e.g. NICE CG95, SIGN 151). If the patient is asymptomatic consider reviewing modifiable cardiovascular risk factors and managing as per guidelines for primary prevention (e.g. NICE CG 181).)* |
| --- |
